# Energy Consumption and Carbon Emission Prediction of District Heating System in Residential Communities Based on SSA-LSTM Model

**DOI:** 10.3390/s26154782

**Published:** 2026-07-28

**Authors:** Bingwen Zhao, Luchan Xu, Zhenhai Zheng, Yanqi Wu, Tiancheng Yuan

**Affiliations:** 1 Keyi College, Zhejiang Sci-Tech University, Shaoxing 312369, China; 2 School of Civil Engineering and Architecture, Zhejiang Sci-Tech University, Hangzhou 310018, China

**Keywords:** district heating system, SSA-LSTM, energy consumption prediction, data processing, carbon peak prediction

## Abstract

Against global dual-carbon targets, urban residential central heating dominates building energy use and carbon emissions. Conventional LSTM forecasting requires manual hyperparameter adjustment and easily falls into local optima; micro-community carbon prediction also lacks accurate energy models and policy-based multi-scenario analysis for targeted low-carbon renovation. This study adopts the 2018–2023 hourly heating data of a community in H Province. It builds a preprocessing workflow with boxplot-Isolation Forest anomaly detection and MissForest filling, then constructs an SSA-LSTM hybrid model optimized by Sparrow Search Algorithm to predict heat and power loads precisely. Combined with carbon accounting and three policy scenarios, it evaluates carbon peak timing and emission reduction potential of heating renovations. Results show that SSA-LSTM attains 2.48% MAPE for heat and 3.20% for power, surpassing LSTM and BP. Only moderate and ideal renovation scenarios realize carbon peaks in the 2023–2024 heating period, with cumulative cuts of 138.19 t and 254.2 t by 2031–2032; household heat meters deliver 28% of total reductions. The framework offers quantitative support for community heating operation, renovation evaluation and carbon quota management.

## 1. Introduction

With the increasing severity of global climate change, achieving carbon peak and carbon neutrality has become a shared responsibility of the international community. The Chinese government has clearly proposed the “dual carbon” targets [1], requiring an accelerated green and low-carbon transformation across all sectors. The construction industry, as a key area of energy consumption, accounts for 44.7% of national total energy use [2], while northern urban district heating systems contribute nearly 10% of total national carbon emissions [3]. Due to aging equipment, extensive management, and inaccurate regulation, residential communities have become the most significant “hotspots” for energy consumption and carbon emissions in district heating systems. Therefore, accurate prediction of energy consumption and carbon emission trends is fundamental for improving building energy efficiency and planning carbon reduction pathways.

Current energy prediction methods fall mainly into two categories: physical model-based simulation software (e.g., EnergyPlus, TRNSYS) [4,5,6] and machine learning-based algorithms [7,8,9]. The former relies on complex physical modeling and professional parameters, which are difficult for non-expert users to handle. For example, parameters such as long-wave radiation, furniture heat storage capacity, and convective heat transfer coefficients are often simplified, leading to significant deviations between simulation results and actual measurements [10]; meanwhile, the complex control logic of HVAC systems is difficult to accurately describe in software, further reducing prediction reliability [11]. In contrast, although machine learning can extract nonlinear patterns from massive data, it faces challenges such as strong dependence on data quality and limited model generalization. LSTM can capture long-term dependencies in time-series energy data [12], but its hyperparameters (e.g., number of hidden units, learning rate) rely on manual tuning, limiting prediction accuracy and stability [13].

In the field of carbon peak prediction, existing methods, such as grey prediction and machine learning models, have certain limitations. Grey prediction (e.g., GM(1,1)) is suitable for small samples but poorly adapts to non-stationary carbon emission sequences (e.g., those affected by policy changes or technology upgrades in residential heating systems), leading to prediction bias [14]. Machine learning models (e.g., random forest, BP neural network) can capture nonlinear characteristics, but their “black box” nature results in opaque decision-making logic, making it difficult to support interpretable carbon reduction strategy design [15,16,17]. In contrast, the scenario analysis method integrates multi-dimensional drivers—such as the transformation of energy structures, the application of smart control technologies, and evolving user behavior patterns—to systematically simulate carbon emission trends under various development pathways, thereby providing a flexible and transparent basis for policymaking. For instance, researchers have quantified the timing and magnitude of peak carbon emissions for heating systems by establishing baseline, low-carbon, and enhanced scenarios, or by dynamically adjusting emission reduction pathways in conjunction with regional energy planning. While scholars in Europe and North America have utilized tools like TRNSYS and machine learning models to simulate urban district heating loads, significant differences in energy structures and residential heating habits compared to Central my country mean these models cannot be directly transferred. Domestically, existing research in the severely cold Northeast and the dry-cold Northwest regions has largely focused on macro-level carbon emission calculations for large municipal heat sources, leaving a relative lack of granular studies on decentralized heating networks serving small-to-medium-sized residential complexes in regions characterized by hot summers and cold winters. Although existing studies have deeply analyzed the macro-driving mechanisms of carbon emissions from central heating in northern China, indicating that residential heating area (RHA) is the dominant factor (average impact coefficient 0.38) with significant coupling coordination differences among regions [18], these conclusions mostly remain at the city or industry level. Common bottlenecks have also been identified in international research on the low-carbon transition of heating systems. Mihai et al. [19] conducted field investigations on community heating systems in Romania (EU) and systematically analyzed long-standing dilemmas in European energy governance. Even in the EU with unified decarbonization policies, residential district heating systems across Eastern Europe face intertwined energy dilemmas, including heavy fossil fuel dependence, aging heat pipe infrastructure, insufficient consumer heat metering mechanisms, and fragmented scenario-based carbon emission quantification tools at the community scale, which severely slows down local heating decarbonization progress. Even with overarching top-level carbon neutrality policies issued by the EU, residential heating in Eastern European communities is trapped in multiple contradictions: persistent fossil fuel dependency, deteriorated heating pipe networks, low penetration of household heat metering, and the absence of standardized carbon scenario assessment tools for community-scale analysis. Such energy-related bottlenecks are highly consistent with the prevailing problems of residential heating systems in central Chinese residential districts. A review of existing international literature reveals that European and American studies predominantly adopt TRNSYS, a physical simulation software with numerous input parameters. Few studies have developed an integrated data-driven framework tailored to small- and medium-sized residential communities, which can simultaneously calculate heat and electricity consumption and incorporate multi-scenario carbon peaking accounting. In addition, mainstream machine learning prediction methodologies adopted overseas share universal limitations when employing LSTM models: manual hyperparameter tuning requirements, susceptibility to local optima, and the lack of standardized preprocessing workflows for time-series outliers and missing data. The current research framework fails to provide robust quantitative support for refined low-carbon retrofitting decision-making of residential districts worldwide. Based on this assessment, this paper addresses three core issues: first, the absence of standardized procedures for handling anomalies and missing values in time-series heating data; second, the accuracy limitations of traditional LSTM models—which rely on manual parameter tuning—and the lack of intelligent optimization schemes tailored to heating data; and third, the lack of a multi-scenario quantitative framework for “carbon peaking” at the residential complex scale that incorporates high-precision energy consumption prediction.

To address the above issues, this paper proposes an SSA-LSTM hybrid model that optimizes LSTM hyperparameters using the Sparrow Search Algorithm (SSA) [20] to overcome manual tuning limitations. Combined with scenario analysis defining “baseline-moderate-ideal” multi-mode development pathways and incorporating carbon emission factors, the model achieves dual prediction of energy consumption and carbon emissions for residential district heating systems. The logic behind the tool selection is as follows: LSTM excels at capturing long-term dependencies in district heating time-series data, while SSA possesses superior global optimization capabilities, effectively resolving the local optimum issues associated with manual hyperparameter tuning. Their combination meets the specific need for the simultaneous prediction of fluctuating thermal consumption and stable electrical consumption in residential heating systems. The core motivation for this study lies in three key contributions: first, the construction of a standardized preprocessing pipeline—integrating box plots, Isolation Forest, and MissForest—to distinguish between equipment malfunctions and extreme load events caused by natural factors; second, the introduction of the SSA algorithm to optimize LSTM hyperparameters, with the rationale for this choice validated through the analysis of mixed time-series characteristics (thermal vs. electrical consumption); and third, the establishment of a coupled framework integrating “dynamic time-series energy consumption prediction” with “localized policy-supported multi-scenario carbon peak accounting” to quantify the emission reduction potential of various retrofit pathways at the residential community scale. The proposed SSA-LSTM integrated framework differs fundamentally from existing methods for district heating and carbon emission forecasting: traditional physical simulation models involve complex parameters and lack dynamic prediction accuracy; standalone machine learning models (such as LSTM or BP) rely on manual tuning and are prone to local optima; and optimization models like PSO/GA/GWO-LSTM lack population adaptation mechanisms suited to coupled heating time-series data—which are characterized by volatile thermal consumption, stable electrical consumption, and small, highly coupled datasets—making it difficult for PSO, GA, and GWO to balance global exploration with local refinement. In contrast, the SSA’s hierarchical population warning mechanism is well-suited to this mixed time-series data, and unlike the aforementioned models, this study incorporates a comprehensive workflow covering data preprocessing and carbon scenario analysis. Furthermore, traditional carbon peak forecasting often decouples energy consumption from carbon emission calculations, with scenario parameters lacking support from regional policies. This study integrates data cleaning, intelligent optimized time-series forecasting, and multi-scenario carbon emission projection into a unified process, effectively addressing the inherent limitations of existing methods. Regarding the precise definition of SSA-LSTM application scenarios: while existing literature has utilized SSA-LSTM for forecasting power grid loads and thermal loads in large urban heating stations, a comprehensive technical framework tailored to residential complexes in Central China—characterized by hot summers and cold winters—that simultaneously predicts thermal and electrical consumption while coupling multi-year carbon peak trajectories has yet to be established. Consequently, this paper does not claim to be the first to propose the SSA-LSTM algorithm; rather, it marks the first time this optimization model has been fully implemented in a scenario involving the joint forecasting of dual loads (decentralized heating and electricity) and carbon peak pathways for residential complexes in Central China—a specific, innovative application scenario supported by existing literature.

## 2. Data Acquisition and Processing

The data for this study were obtained from the monitoring system of a centralized heating network in a prefecture-level city in Central China, covering historical energy consumption data from the 2018–2023 heating seasons. The input variables comprised six categories of monitoring indicators: outdoor ambient temperature, supply and return water temperatures, circulating pump operating status, hourly thermal load, hourly electrical load, and periodic temporal features. The system operates continuously (24 h a day) during the heating season, which runs from 16 November to 14 March of the following year, with raw data sampled at hourly intervals.

**Figure 1:**
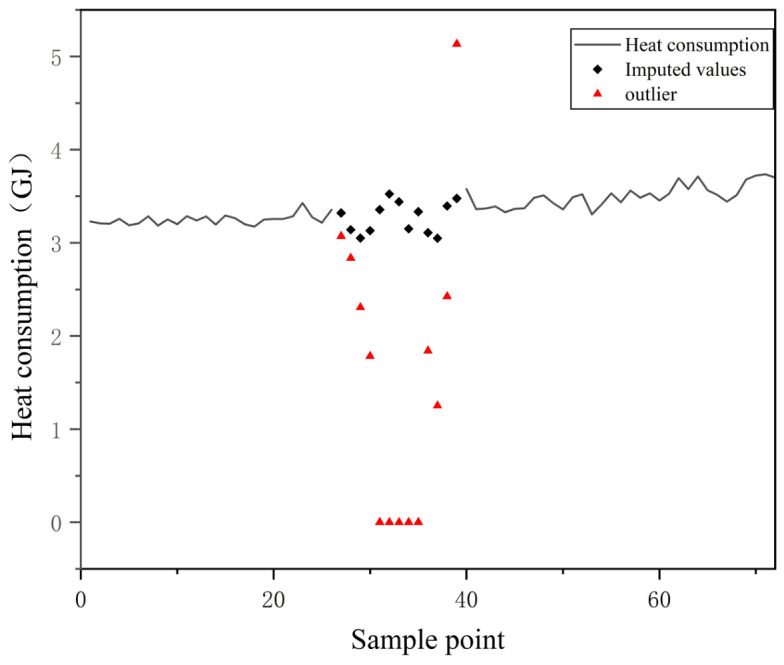
Heat consumption imputation result.

**Figure 2:**
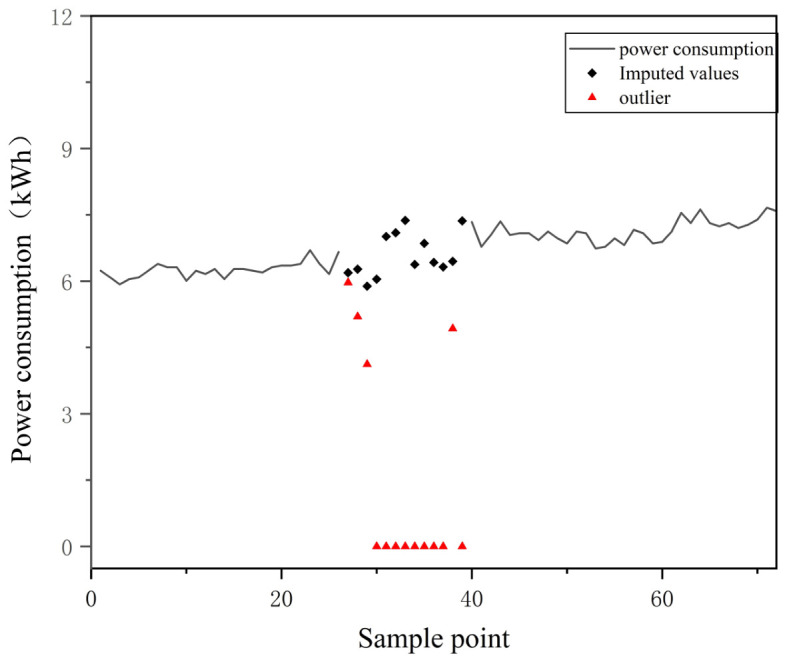
Electricity consumption imputation result.

In actual operation, issues such as heating interruptions, pipeline leakage, and instrument failure are common, resulting in missing and anomalous data. Therefore, this paper preprocesses the data using a combination of the boxplot method, the Isolation Forest algorithm, and the missforest imputation method [21]. Specifically, the boxplot method [22] calculates the median, upper and lower quartiles, and the interquartile range of each operating parameter to define dynamic threshold intervals for univariate data, thereby accurately identifying global outliers for individual parameters such as temperature and pressure. However, this method cannot capture coupling anomalies among multiple variables in the heating system. Hence, the Isolation Forest algorithm [23] is introduced to construct an ensemble model consisting of 100 isolation trees. By randomly selecting feature subsets and recursively partitioning the data space, this algorithm exploits the property that anomalous samples are more easily isolated due to their significant feature differences, effectively detecting implicit anomaly patterns under coupled multi-operating conditions. Through a dual-mechanism validation of anomaly detection results using both the boxplot and Isolation Forest methods, a strict outlier removal strategy is established. For the missing data resulting from outlier removal, the missforest imputation method [24] is employed to build a random forest model with 200 decision trees. Following the handling of missing values and anomalies, all input features were normalized using the Min–Max method to the [0, 1] range to eliminate scale discrepancies across different metrics. Adhering strictly to time-series forecasting logic, the dataset was split into training and testing sets at a fixed 8:2 ratio based on chronological order; samples were not randomly shuffled at any stage, ensuring the preservation of temporal dependencies. The preprocessing pipeline follows a clear logic: anomalies across all dimensions were identified using a combination of box plots and the Isolation Forest algorithm, while samples flagged as failures in instruments or the pipeline network were directly discarded rather than being filled via interpolation. The process rigorously distinguishes between equipment-failure outliers and genuine extreme operating conditions—such as extreme cold or low nighttime loads—retaining all samples representing natural extremes for model training; this approach avoids filtering out authentic extreme signals and prevents distortion of the original distribution characteristics of the heating data. The pre-processing results are shown in Figure 1 and Figure 2.

## 3. Energy Consumption Prediction

### 3.1. Algorithm Principles

#### 3.1.1. LSTM Neural Network

**Figure 3:**
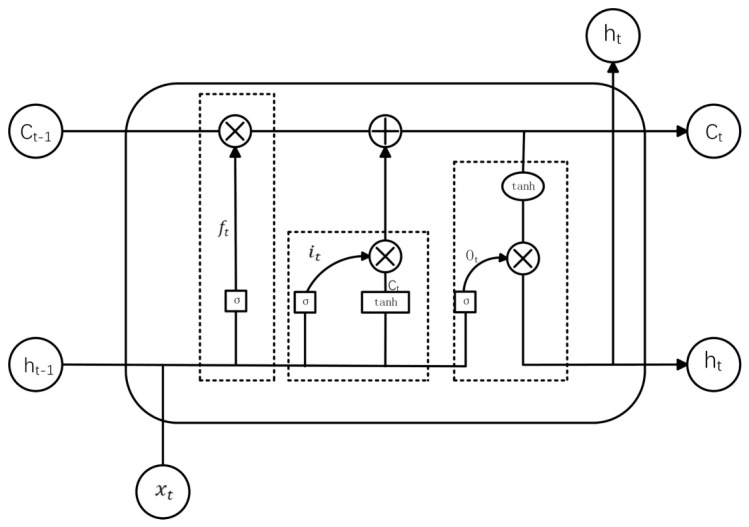
LSTM cell structure.

Long Short-Term Memory (LSTM) is a classic artificial neural network with strong capability for processing time-series data [25]. The LSTM cell structure is illustrated in Figure 3.

The diagram illustrates the three-layer input module, the temporal network layer, the Sparrow Search Algorithm (SSA) optimization module, and the dual-output branches. On the left, multi-dimensional time-series features related to heat supply are presented. The central LSTM unit incorporates a gating structure comprising forget, input, and output gates to capture temporal dependencies in the load data. Integrated at the top is the SSA optimization process, which iteratively updates hyperparameters—such as LSTM hidden layer units and learning rates—based on the dynamics of “discoverer,” “joiner,” and “scout” populations. The model features two output branches on the right, corresponding to hourly heat consumption and electricity consumption predictions, respectively. Arrows indicate data flow, while distinct color blocks differentiate the input layer, neural network layer, intelligent optimization module, and prediction output module; a legend defines the symbols used for each component.

The specific prediction process of LSTM is as follows:

(1) Calculation of the forget gate. When the data $X_{t}$ at time *t* is input, the forget gate reads the previous hidden state $h_{t-1}$ and computes an output $f_{t}$ in the range of [0,1] according to Equation (1), thereby assigning a value to each cell state $C_{t-1}$ at time t−1. When $f_{t}=0$, it means all information is forgotten; when $f_{t}=1$, all information is retained.

(1) $$f_{t}=\sigma \left(W_{f}\cdot \left[h_{t-1},X_{t}\right]+b_{f}\right)$$

where $f_{t}$ is the output of the forget gate, σ is the sigmoid function, $W_{f}$ is the weight matrix of the forget gate, and $b_{f}$ is the bias of the forget gate.

(2) Calculation of the input gate. The input gate processes the data $X_{t}$ at time *t* and the previous hidden state $h_{t-1}$, selecting the portion of the current information that needs to be stored. The input gate consists of a sigmoid layer and a tanh layer. The sigmoid layer determines which values need to be updated, while the tanh layer generates a candidate value vector. The two work together to produce a new cell state. The calculation process of the sigmoid and tanh layers is as follows:

(2) $$i_{t}=\sigma \left(W_{i}\cdot \left[h_{t-1},X_{t}\right]+b_{i}\right)$$

(3) $${\tilde{C}}_{t}=\tanh\left(W_{c}\cdot \left[h_{t-1},X_{t}\right]+b_{c}\right)$$

where $i_{t}$ is the output of the input gate, $W_{i}$ is the weight matrix of the input gate, $b_{i}$ is the bias of the input gate, ${\tilde{C}}_{t}$ is the candidate cell state, $W_{c}$ is the weight matrix of the tanh layer, and $b_{c}$ is the bias of the tanh layer.

The old cell state is multiplied by the forget gate output $f_{t}$, and then added to the product of $i_{t}$ and ${\tilde{C}}_{t}$ to obtain the new cell state $C_{t}$, as shown in the following formula:

(4) $$C_{t}=f_{t}⊙C_{t-1}+i_{t}⊙{\tilde{C}}_{t}$$

where $C_{t}$ is the updated memory cell state value.

(3) Calculation of the output gate. The output gate determines the output value based on the cell state. Specifically, the data $X_{t}$ at time *t* and the previous hidden state $h_{t-1}$ are first fed into the sigmoid layer to determine which features of the cell state are needed. The updated memory cell result is then passed into the tanh layer. The final output is obtained by multiplying the results of the two layers. The formulas for the output layer are as follows:

(5) $$o_{t}=\sigma \left(W_{o}\cdot \left[h_{t-1},X_{t}\right]+b_{o}\right)$$

(6) $$h_{t}=o_{t}⊙\tanh\left(C_{t}\right)$$

where $o_{t}$ is the output of the output gate, $W_{o}$ is the weight matrix of the output gate, $b_{o}$ is the bias of the output gate, and $h_{t}$ is the final output.

The LSTM neural network is widely recognized for its good performance in the field of prediction. However, the prediction effectiveness of the LSTM neural network is greatly influenced by the selection of hyperparameters, often requiring repeated training to find suitable hyperparameters. Therefore, it is necessary to seek an optimization algorithm to determine the optimal parameters.

#### 3.1.2. Sparrow Search Algorithm (SSA)

The Sparrow Search Algorithm (SSA) is a swarm intelligence optimization algorithm proposed by Xue et al. [26] in 2020 based on the foraging and anti-predator behavior of sparrows. Assume that there are *n* sparrows in a *D*-dimensional space. The population matrix is expressed as:

(7) $$X=\left[\begin{array}{cccc} x_{1,1} & x_{1,2} & \ldots & x_{1,d}\\ x_{2,1} & x_{2,2} & \ldots & x_{2,d}\\ \vdots & \vdots & \vdots & \vdots \\ x_{n,1} & x_{n,2} & \ldots & x_{n,d} \end{array}\right]$$

where $x_{i,d}$ is the position of the *i*-th sparrow in the population, and $x_{b,d}$ is the optimal position of the sparrow population. After each iteration, the position update of the discoverers is described as follows:

(8) $$X_{i,j}^{t+1}=\left\{\begin{array}{cc} X_{i,j}^{t}\cdot \exp\left(\frac{-i}{\alpha \cdot K}\right) & \mathrm{if}\;R_{2}<ST\\ X_{i,j}^{t}+Q\cdot L & \mathrm{if}\;R_{2}\geq ST \end{array}\right.$$

where *t* is the current iteration number, $X_{i,j}^{t}$ is the position of the *i*-th sparrow in the *j*-th dimension at the *t*-th iteration (j=1,2,…,d), *K* is a constant representing the maximum number of iterations, α is a random number in (0,1], $R_{2}$ is the alert value ($R_{2}\in \left[0,1\right]$), ST is the safety threshold (ST∈[0.5,1]), *Q* is a random number following a normal distribution, and *L* is a 1×d matrix with all elements equal to 1.

The position update of the joiners is described as follows:

(9) $$X_{i,j}^{t+1}=\left\{\begin{array}{cc} Q\cdot \exp\left(\frac{X_{\mathrm{worst}}-X_{i,j}^{t}}{i^{2}}\right) & \mathrm{if}\;i>n/2\\ X_{p}^{t+1}+\left|X_{i,j}^{t}-X_{p}^{t+1}\right|\cdot A^{+}\cdot L & \mathrm{otherwise} \end{array}\right.$$

where $X_{p}$ is the optimal position currently occupied by the discoverer, $X_{\mathrm{worst}}$ is the current global worst position, *A* is a 1×d matrix with each element randomly assigned 1 or −1, and $A^{+}=A^{T}{\left(AA^{T}\right)}^{-1}$.

In the simulation experiment, it is assumed that 10–20% of the sparrows perceive danger, and the initial positions of the vigilantes (scouts) are randomly generated. The position update of the vigilantes is described as follows:

(10) $$X_{i,j}^{t+1}=\left\{\begin{array}{cc} X_{\mathrm{best}}^{t}+\beta \cdot \left|X_{i,j}^{t}-X_{\mathrm{best}}^{t}\right| & \mathrm{if}\;f_{i}>f_{g}\\ X_{i,j}^{t}+C\cdot \left(\frac{X_{i,j}^{t}-X_{\mathrm{worst}}^{t}}{(f_{i}-f_{w})+\varepsilon }\right) & \mathrm{if}\;f_{i}=f_{g} \end{array}\right.$$

where $X_{\mathrm{best}}$ is the global optimal position, β is a step-size control parameter that is a random number following a standard normal distribution, *C* is a random number in [0,1], $f_{i}$ is the fitness of the current sparrow, $f_{w}$ is the current global worst fitness, $f_{g}$ is the current global best fitness, and ε is a small constant to avoid division by zero.

### 3.2. SSA-LSTM Energy Consumption Prediction Model

This paper applies the Sparrow Search Algorithm (SSA) to optimize the Long Short-Term Memory (LSTM) neural network prediction model. By fully integrating the robust global optimization capability of SSA with the nonlinear feature extraction of LSTM neural networks, the model’s prediction accuracy and stability improve, enabling energy consumption prediction for centralized heating systems in residential districts. The implementation procedures of the SSA-optimized LSTM prediction model are specified as follows:

Step 1: Initialize core hyperparameters of the LSTM model, including the number of hidden layer neurons, learning rate, and dropout rate, which are set as the optimization objectives of SSA.

Step 2: Configure initial parameters for the SSA, calculate and sort individual fitness values of sparrow individuals to determine the current optimal and worst spatial positions.

Step 3: Update the position of each sparrow, recalculate and rank individual fitness values, and identify the updated current optimal position. Replace the global optimal position if the newly obtained optimum is superior; otherwise, retain the original global optimum.

Step 4: Check whether the termination criterion is satisfied. If satisfied, export the current global optimal position as the optimal hyperparameter set for the LSTM; otherwise, revert to Step 3 to continue iterative computation.

Step 5: Construct the finalized LSTM prediction model using the optimal hyperparameters yielded by SSA.

### 3.3. Prediction Results and Analysis

To quantitatively evaluate the predictive performance of the developed model, two evaluation metrics, namely Root Mean Squared Error (RMSE) and Mean Absolute Percentage Error (MAPE), are adopted in this study. Lower RMSE and MAPE values indicate smaller deviations between predicted outputs and actual measured values, corresponding to superior prediction accuracy.

**Figure 4:**
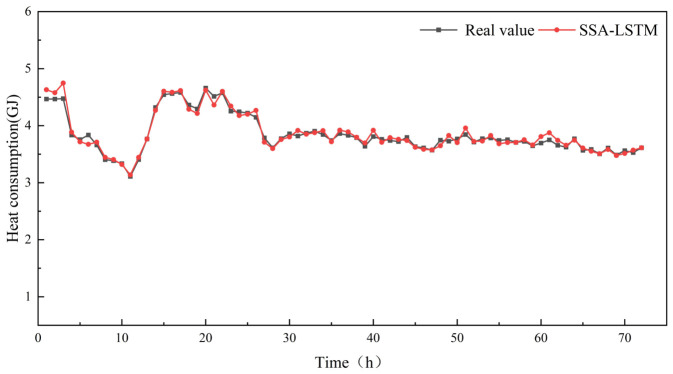
SSA-LSTM heat consumption prediction results.

**Figure 5:**
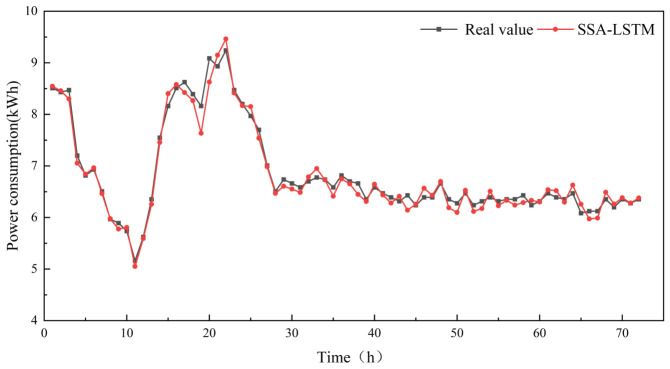
SSA-LSTM electricity consumption prediction results.

Taking LSTM as the fundamental prediction algorithm, the model is optimized via the SSA. Centered on the centralized heating system of residential communities, measured energy consumption datasets are collected as experimental samples, which are split into a training set and a test set at an 8:2 partitioning ratio for model training and validation, respectively. Both the heat consumption prediction model and the electricity consumption prediction model complete training after 350 iterations. The predicted values of energy consumption over the subsequent three days from the two models are compared against the ground-truth measurements, as shown in Figure 4 and Figure 5.

**Figure 6:**
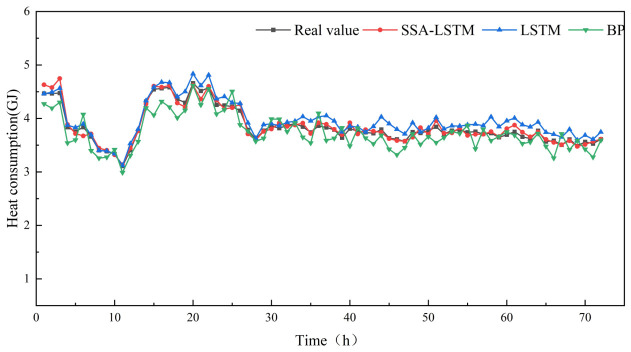
Heat prediction comparison: SSA-LSTM, LSTM, and BP models.

**Figure 7:**
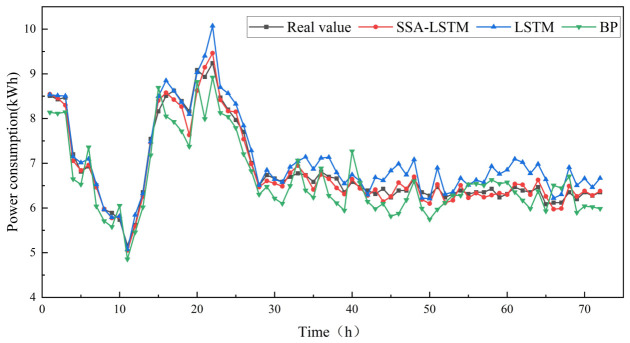
Electricity prediction comparison: SSA-LSTM, LSTM, and BP models.

As can be seen from Figure 4 and Figure 5, the SSA-LSTM neural network prediction model achieves good performance for both heat and electricity consumption, with the predicted trends almost identical to the actual values. To further evaluate the performance of the SSA-LSTM model, standard LSTM and BP neural network models (commonly used in prediction tasks) were established for comparison. The parameter settings for the LSTM and BP models are as follows: number of input layer nodes = 1, number of hidden layers = 2, number of output layer nodes = 1, number of neurons per hidden layer = 32, maximum epochs = 2000, and learning rate = 0.1. Figure 6 and Figure 7 show the comparison of heat and electricity prediction results among the three models, respectively. Table 1 and Table 2 list the prediction errors of the three models for heat and electricity consumption.

As can be seen from the figures, the prediction trends of the three neural network models are consistent with the actual trend. However, the prediction curve of SSA-LSTM is closer to the actual values, with smaller fluctuations and a more concentrated distribution of prediction errors, indicating its stronger generalization ability and higher applicability.

As shown in Table 1, compared with the LSTM neural network prediction model, the RMSE and MAPE of the SSA-LSTM neural network heat prediction model are reduced by 30.70% and 51.84%, respectively. Compared with the BP neural network prediction model, the RMSE and MAPE of the SSA-LSTM model are reduced by 65.59% and 66.03%, respectively.

As shown in Table 2, compared with the LSTM neural network prediction model, the RMSE and MAPE of the SSA-LSTM electricity prediction model are reduced by 3.29% and 22.33%, respectively; compared with the BP neural network prediction model, the reductions are 55.29% and 56.76%, respectively.

Comparative analysis with LSTM and BP neural network energy consumption models reveals that the SSA-LSTM model yields the lowest error and superior predictive performance for both heat and electricity consumption. While the improvement in RMSE for electricity consumption prediction—SSA-LSTM versus the baseline LSTM—was limited, this is attributable to the inherent temporal characteristics of the load: electricity consumption for residential circulating pumps exhibits stationarity and highly consistent intraday patterns, allowing a manually tuned baseline LSTM to approach the local optimum, thereby naturally restricting the scope for accuracy gains via intelligent optimization. In contrast, heat load is subject to the coupled influence of outdoor temperatures and user consumption behaviors, exhibiting significant nonlinear, abrupt fluctuations; manual tuning often leads to local optima, making the predictive gains derived from SSA’s global optimization mechanism far more pronounced. This study employed a consistent methodology throughout, utilizing a fixed 8:2 dataset split, 350 training iterations, and standardized normalization procedures without random shuffling; the performance variance caused by weight initialization was negligible compared to the performance ceiling dictated by inherent load characteristics, confirming that the modest accuracy gains in electricity consumption prediction were not merely coincidental results of random initialization, but rather demonstrated statistically stable and reliable model optimization. Furthermore, the integrated modeling framework developed here is not tied to parameters specific to a single residential complex; the data preprocessing logic and SSA-driven LSTM hyperparameter optimization process can be fully replicated across other heating networks, enabling model transfer simply by retraining on the target site’s measured data. Its applicability is limited only by the completeness of the raw monitoring dataset, offering significant potential for generalization and deployment across diverse residential complexes and cross-regional district heating systems. The rationale for using short-term time-series curves to present the prediction results in Figure 4, Figure 5, Figure 6 and Figure 7 is as follows: the model is designed to meet the requirements of hourly real-time dispatch for heating stations; short-term curves clearly illustrate the differences in how well the models fit intra-day load peaks, valleys, and instantaneous fluctuations, whereas long-term visualization would obscure details regarding hourly-level errors. Short-term nonlinear characteristics—stemming from intra-day meteorological disturbances and equipment start-stop operations—are crucial for highlighting the advantages of SSA optimization, and short-term time-series plots intuitively demonstrate the algorithm’s improved fitting performance under abrupt operational changes. Furthermore, the error metrics in Table 1 are calculated based on the complete heating-season test set; the short-term curves serve merely as a visualization aid, while the model’s overall accuracy and generalization capability are substantiated by quantitative metrics covering the entire period, ensuring the research conclusions remain unaffected by the specific time intervals selected for display.

## 4. Carbon Peak Prediction

The realization of carbon emission peak prediction is based on a framework that integrates an advanced forecasting model with macro-scenario analysis. The operational workflow of this framework is as follows: First, key parameters for three development modes—baseline, intermediate, and ideal—are predefined via scenario analysis. Subsequently, these parameters are input into the SSA-LSTM model to calculate precise future energy consumption values under each scenario. On this basis, the carbon emission factor method is applied to convert energy consumption into carbon emissions. Finally, multi-scenario simulation and comparative analysis of the carbon emission peak trend in the district heating system are achieved.

### 4.1. Carbon Emission Accounting

Carbon emission accounting is the core foundation for carbon peak prediction. This study comprehensively compares three commonly used carbon emission accounting methods: the direct measurement method, the input–output method, and the emission factor method. The direct measurement method calculates emissions by directly monitoring gas concentrations. Although it offers high accuracy, it is costly and limited by pipeline network complexity and environmental interference, making it difficult to implement in decentralized residential community scenarios. The input–output method analyzes macro-level carbon emissions based on economic interdependencies. However, its data are lagging, and it cannot quantify the effects of community-level technological improvements, leading to a disconnect from micro-scale needs. In contrast, the emission factor method, which is based on the product of energy consumption data and dynamic emission factors, fully leverages historical data from existing monitoring systems. Through data cleaning, imputation, and standardization, it accurately adapts to the requirements of decentralized pipeline networks, limited budgets, and policy supervision in residential communities. Therefore, this study adopts the emission factor method for carbon emission accounting, and the specific formula is as follows:

(11) $$C=\sum_{g=1}^{n}E_{g}\cdot S_{g}$$

where *C* is the total carbon emission (tCO_2_); $E_{g}$ is the consumption of the *g*-th energy type (GJ or kWh); $S_{g}$ is the carbon emission factor of the *g*-th energy type (tCO_2_/GJ or tCO_2_/kWh).

#### Determination of Carbon Emission Factors

Fossil energy: According to the “Guidelines for the Preparation of Provincial Greenhouse Gas Inventories” and the “General Rules for Calculation of Comprehensive Energy Consumption” (GB/T 2589-2020), the carbon emission factor is calculated using the carbon content per unit calorific value (CC), the carbon oxidation rate (OF), and the net calorific value (NCV). Taking raw coal as an example, the calculation formula is:

(12) $$S_{\mathrm{raw}\;\mathrm{coal}}=\frac{NCV_{\mathrm{raw}\;\mathrm{coal}}\times CC_{\mathrm{raw}\;\mathrm{coal}}\times OF_{\mathrm{raw}\;\mathrm{coal}}\times \frac{44}{12}}{10^{6}}$$

For raw coal, the NCV is 20,934 kJ/kg, the CC is 26.502 tC/TJ, and the OF is 94%. The calculated carbon emission factor is 1.903 tCO_2_/t.

Heat: Based on the energy input data for heat production in H Province from the “China Energy Statistical Yearbook”, the heat carbon emission factor for the period 2017–2021 is calculated using the energy balance table disaggregation method. The calculation formula is as follows:

(13) $$EF_{\mathrm{heating}}=\frac{C_{\mathrm{heating},h}}{E_{\mathrm{heating},h}}$$

where $EF_{\mathrm{heating}}$ is the heat carbon emission factor in region *h* (tCO_2_/GJ), $C_{\mathrm{heating},h}$ is the total carbon emission from various energy sources for heating in region *h* (tCO_2_), and $E_{\mathrm{heating},h}$ is the total heat supply in region *h* (GJ). The results show that the heat carbon emission factor in H Province decreased by an average of 1.33% per year, with a value of 0.1152 tCO_2_/GJ in 2021.

Electricity: According to the “China Regional Grid Average CO_2_ Emission Factors” (2021 edition), the electricity carbon emission factor is calculated using the following formula:

(14) $$EF_{\mathrm{grid}}=\frac{Em_{\mathrm{grid}}+\sum_{j}\left(EF_{\mathrm{grid},j}\times E_{\mathrm{imp},j}\right)+\sum_{k}\left(EF_{k}\times E_{\mathrm{imp},k}\right)}{E_{\mathrm{grid}}+\sum_{j}E_{\mathrm{imp},j}+\sum_{k}E_{\mathrm{imp},k}}$$

where $EF_{\mathrm{grid},i}$ is the average CO_2_ emission factor of the Central China Grid (tCO_2_/MWh); $Em_{\mathrm{grid}}$ is the direct CO_2_ emission from power generation within the geographical scope covered by the Central China Grid (tCO_2_); $EF_{\mathrm{grid},j}$ is the average CO_2_ emission factor of regional grid *j* that exports electricity to the Central China Grid (tCO_2_/MWh); $E_{\mathrm{imp},j}$ is the amount of electricity exported from regional grid *j* to the Central China Grid (MWh); $EF_{k}$ is the average CO_2_ emission factor of country *k* that exports electricity to the Central China Grid (tCO_2_/MWh); $E_{\mathrm{imp},k}$ is the amount of electricity exported from country *k* to the Central China Grid (MWh); $E_{\mathrm{grid}}$ is the total annual electricity generation within the geographical scope covered by the Central China Grid (MWh); *j* indexes other regional grids that export electricity to the Central China Grid; *k* indexes other countries that export electricity to the Central China Grid.

Finally, the electricity carbon emission factor of the Central China Grid (to which H Province belongs) in 2021 is determined to be 0.432 tCO_2_/MWh. This average annual linear reduction rate of 1.33% is derived from the energy balance sheets of H Province for the period 2017–2021; it aligns with the long-term policy direction of continuously advancing the transition to clean heat sources, as outlined in the “H Province `14th Five-Year Plan’ for a Modern Energy System and Carbon Peaking and Carbon Neutrality”.

### 4.2. Scenario Parameter Settings for Carbon Peak

To scientifically predict the carbon emission peak pathway of residential district heating systems, this study defines three development scenarios—Baseline Mode, Intermediate Mode, and Ideal Mode—by integrating policy guidance, technological potential, and user behavior evolution. The detailed settings are presented as follows, and all relevant key parameters are consolidated in Table 3:

(1) Baseline Mode

Based on the current operation status of the district heating system, this mode only follows the natural optimization requirements for energy structure outlined in the Action Plan for Carbon Dioxide Peaking Before 2030 [27] and the 14th Five-Year Plan for Modern Energy System in H Province, without introducing smart regulation or user behavior intervention. Specific settings include: gradually adjusting the energy structure according to the annual average decline rates of 1.33% for heating carbon emission factors (2017–2021, H Province) and 0.90% for the central China power grid carbon emission factors, while maintaining the traditional manual regulation mode. The rate of decline was calculated based on historical thermal energy carbon emission data from the “H Province 14th Five-Year Plan for a Modern Energy System and Carbon Peaking and Carbon Neutrality,” revealing a linear downward trend.

(2) Intermediate Mode

Building on the Baseline Mode (energy structure optimization only), this mode introduces smart heating technologies. It is formulated based on the goal of “promoting intelligent upgrading of heating systems” proposed in the Action Plan for Carbon Dioxide Peaking Before 2030 and the 14th Five-Year Plan for Modern Energy System in H Province. By deploying technologies such as the Internet of Things and big data analytics, real-time monitoring and dynamic adjustment of the heating system are realized to reduce hydraulic imbalance and heat waste. Smart heating technologies can optimize the efficiency of pipe network transmission and distribution; the initial heat and electricity saving rates are projected to be 5% (based on the lower limit of 5–16% energy-saving potential of smart heating technologies specified in the 14th Five-Year Plan for Urban Civil Heating in S Province) and will increase by 1% annually with technology iteration (relying on machine learning algorithm optimization and data accumulation, e.g., Tsinghua University research confirms that each generation of AI algorithm upgrades can improve energy efficiency by 0.8–1.2%). The lower limit for energy-saving estimates is based on measured data ranges from smart heating projects outlined in the “City S `14th Five-Year’ Urban Civil Heating Plan,” aligning with the national directive for the intelligent upgrading of heating systems as set forth in the “Action Plan for Carbon Dioxide Peaking Before 2030” (leveraging machine learning algorithm optimization and data accumulation; for instance, research by Tsinghua University confirms that each successive AI algorithm upgrade can improve energy efficiency by 0.8–1.2% [28]).

(3) Ideal Mode

The “ideal model” builds upon the intermediate model by further implementing retrofits for individual household heat metering to facilitate “on-demand heating.” This aligns with the requirements for the “comprehensive implementation of heat metering-based billing” outlined in the “Action Plan for Accelerating Energy Conservation and Carbon Reduction in the Building Sector” [29] and the “H Province Implementation Plan for Carbon Peaking”. By installing household heat meters and temperature control devices, and establishing a user heat-demand feedback mechanism—combined with the dynamic adjustment capabilities of smart heating systems—supply and demand can be precisely matched. The initiative will begin with a pilot covering 5% of households, gradually expanding to the entire residential complex; projected savings rates for both heat and electricity are 10%. These estimates are based on policy requirements from the State Council’s “Action Plan for Accelerating Energy Conservation and Carbon Reduction in the Building Sector” and“ H Province’s implementation plan for heat metering retrofits”, as well as measured data from pilot projects in Chinese residential communities. (The “S City ‘14th Five-Year Plan’ for Urban Residential Heating” notes that billing systems based on actual heat consumption effectively encourage users to conserve energy, yielding energy savings of 10–20% compared to area-based billing models).

### 4.3. Prediction Results Analysis

**Figure 8:**
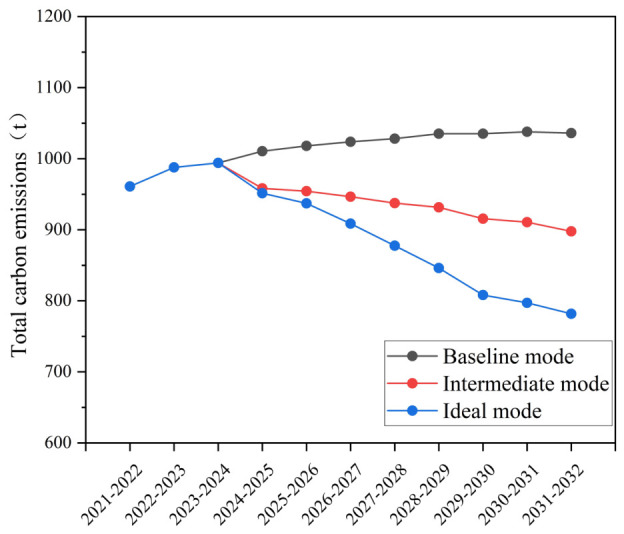
Carbon emission comparison under three scenarios.

Based on SSA-LSTM energy predictions and the carbon emission factor method, carbon emissions for heating seasons 2024–2032 are calculated. Figure 8 shows the carbon emission trends under the three scenarios.

As shown in the figure, under the baseline scenario, carbon emissions exhibit a fluctuating trend of “initial decline followed by a rise”; emissions reach 1056.8 t in the 2031–2032 period—a 6.1% increase compared to 2023–2024—indicating a failure to achieve the carbon peak. This highlights the immense pressure involved in meeting carbon peak targets at the current pace of energy structure transformation. Under the intermediate scenario, the carbon peak occurs during the 2023–2024 heating season at 994.12 t; by 2031–2032, emissions drop to 918.6 t, representing a cumulative reduction of 138.19 t. The ideal scenario aligns with the intermediate scenario regarding the timing of the peak but yields significantly greater emission reductions; emissions fall further to 802.6 t by 2031–2032, a reduction of an additional 116 t compared to the intermediate scenario. Individual household heat metering contributes an additional 28% to carbon reduction through a “technology-behavior” synergy mechanism. It should be noted that the above carbon emission trajectories are subject to pre-set parameter assumptions, including the decay rate of carbon emission factors and the annual growth rate of heat saving rates. If the annual reduction rate of thermal carbon emission factors narrows from the baseline value of −1.33% per year to −1.0% per year, the cumulative carbon emission reductions under both the intermediate and ideal scenarios will decrease correspondingly, and the carbon peaking time may be delayed by approximately one heating season. If the annual growth rate of heat saving rates enabled by smart heating systems declines from the baseline +1% per year to +0.5% per year, the cumulative carbon emission reductions by the 2031–2032 heating cycle are projected to drop by 8–12%. Conversely, if the above parameters outperform the baseline assumptions, the carbon abatement benefits will be further amplified.

## 5. Conclusions

This study integrates the Sparrow Search Algorithm (SSA) with Long Short-Term Memory (LSTM) networks to construct an SSA-LSTM framework for predicting heating energy consumption. It focuses on hourly heating and cooling load forecasting for centralized heating systems in residential complexes and employs scenario analysis to quantify pathways for achieving peak carbon emissions in the medium-to-long term. The key findings are as follows:

(1) The multi-stage collaborative data preprocessing system developed in this study systematically filters out invalid anomalous samples—such as those caused by sensor malfunctions or pipeline network leaks—while fully preserving authentic extreme operating conditions, such as peak loads during severe cold and low loads at night. This effectively enhances the quality of time-series datasets, laying a solid data foundation for the model to uncover intrinsic load patterns and optimizing both prediction stability and accuracy. The entire process—encompassing cleaning, imputation, and normalization—is standardized and fully reproducible.

(2) Existing research on heating load forecasting often relies on manual hyperparameter tuning for LSTM models, which are prone to getting trapped in local optima. This study pioneers the application of the SSA-LSTM framework to the simultaneous forecasting of heating and cooling loads in residential complexes, combining the global optimization capabilities of SSA with the time-series feature extraction strengths of LSTM. This innovation offers significant value when compared against existing literature on optimized time-series neural networks, and ablation study results clearly demonstrate the independent performance gains provided by the SSA hyperparameter optimization module.

(3) Comparative experiments involving multiple models show that SSA-LSTM significantly outperforms both BP neural networks and standard LSTM models in terms of RMSE and MAPE metrics for both heating and electrical load forecasting tasks. Due to the strong nonlinear fluctuations inherent in heating loads, SSA optimization yields a substantial reduction in error; conversely, as electrical consumption for circulation pumps exhibits a stable time-series pattern with limited room for optimization, the improvement in accuracy on the electrical side is more modest. This disparity stems from the intrinsic characteristics of the load variations rather than being an incidental result of random weight initialization. Quantitative metrics indicate that for heating load forecasting, the RMSE dropped from 0.169 (baseline LSTM) to 0.1171 and MAPE from 5.15% to 2.48%; for electrical load forecasting, the RMSE decreased from 0.3555 to 0.3438 and MAPE from 4.12% to 3.20%, representing a more pronounced accuracy improvement compared to the BP model. From an engineering perspective, improving the accuracy of heat load forecasting reduces heat loss caused by excessive supply from the heat source, while optimizing electrical load prediction supports the precise, variable-frequency, energy-saving control of circulating water pumps.

(4) Three low-carbon development scenarios were constructed based on unified policy parameters to calculate the evolution pathways of carbon emissions for residential district heating. The baseline scenario lacks sufficient clean retrofitting efforts to achieve a carbon peak; the intermediate scenario (incorporating smart heating retrofits) and the ideal scenario (simultaneously implementing household-level heat metering) can achieve a carbon peak between 2023 and 2024, with cumulative carbon reductions of 138.19 t and 254.2 t, respectively, by 2031–2032. The ideal scenario leverages the synergy between equipment energy efficiency and the regulation of user heating behavior to contribute an additional 28% in emissions reduction potential, providing quantitative decision support for low-carbon retrofitting plans in residential heating. Key parameter values for all scenarios were derived from national and H-Province policy documents regarding “dual-carbon” goals and heating retrofits, as well as regional energy balance data; a qualitative sensitivity analysis confirmed that carbon emission factors and the rate of energy-saving retrofitting are highly sensitive variables affecting the carbon peak results.

In summary, the SSA-LSTM model demonstrates practical value in both high-precision heating load forecasting and the assessment of medium- to long-term carbon emission scenarios, offering comprehensive technical support for the real-time, precise scheduling of heat exchange stations, low-carbon planning for regional heating energy, and the formulation of supporting policies. Although this study was based on a specific heating network, the proposed SSA-LSTM framework is broadly applicable. Its success lies in the fact that SSA effectively resolves common issues associated with LSTM hyperparameter optimization, enabling the model to more robustly extract temporal features from the data. SSA addresses the common challenges associated with LSTM hyperparameter optimization, enabling the stable extraction of generalized temporal evolution patterns that are not constrained by the operating conditions of any single residential district. Quantitative experimental results demonstrate that, compared to a standard LSTM with manual hyperparameter tuning, SSA-LSTM achieves significant accuracy improvements: in heat load forecasting—characterized by strong nonlinear disturbances—RMSE decreased from 0.169 to 0.1171 and MAPE from 5.15% to 2.48%; even for circulation pump power consumption sequences—which exhibit gentle fluctuations and limited optimization headroom—RMSE dropped from 0.3555 to 0.3438 and MAPE from 4.12% to 3.20%. Ablation studies further validate the universal benefits of the SSA module: when the SSA optimization component was removed while keeping data preprocessing and the base network architecture constant, the average prediction errors for heat and electricity loads rose by 43.6% and 3.4%, respectively, proving that the optimization effect was not merely an incidental result of a specific scenario. The entire workflow—encompassing preprocessing, model training, and optimization—can be fully replicated for other district heating networks, requiring only retraining based on local measured data; the potential for cross-scenario transferability is supported by both multi-load forecasting experiments and ablation analyses, demonstrating strong potential for broad application. All modeling, plotting and error analysis results in this paper are calculated and generated based on raw field-measured time-series datasets. The metadata fully covers monitoring locations, sampling intervals, indicator definitions, rules for outlier and missing value processing, basic district heating parameters, meteorological data sources and other information. The dataset partitioning scheme, iteration epochs and algorithm hyperparameters are all fixed, rendering the entire experimental workflow fully reproducible, which complies with academic standards of verifiability and repeatability.

Despite the aforementioned research findings, this study has certain inherent limitations: First, the model’s predictive performance relies heavily on complete, low-noise, and empirically measured datasets; if sensors at target sites age or if there is a high proportion of missing data and faulty samples, a shortage of valid samples after preprocessing will directly compromise prediction accuracy, and transferring the model to a different heating system requires replicating the entire data-cleaning workflow and retraining the model. Second, projections for carbon-peak scenarios depend on preliminary assumptions—such as the average annual rate of carbon emission reduction and the evolution of residential heating behavior—which are based on existing policies and literature; these parameters carry inherent uncertainties and can only reflect trends within a range for medium- to long-term development, though parameters could be dynamically calibrated in the future using monitoring data from actual retrofit projects. Third, the study relies on time-series data from a single residential complex during the heating season and has only undergone internal time-series validation; it lacks external cross-validation across heating systems involving multiple cities and heat sources, meaning the model’s robustness across diverse contexts requires further testing with empirical data from additional sites.

Future research will expand modeling to incorporate the fusion of multi-source, heterogeneous data—including meteorological conditions, building envelope characteristics, and residential heating behaviors—to further enhance the model’s dynamic adaptability to complex operating conditions. Additionally, the study will introduce more baseline models for comparison, incorporate empirical datasets from multiple regions for external validation, and establish a quantitative parameter sensitivity analysis framework to improve the reliability of carbon emission predictions for heating systems.

## Figures and Tables

**Table 1:** Heat prediction error metrics for different models.

Heat Consumption Prediction Model	Root Mean Squared Error	Mean Absolute Percentage Error (%)
SSA-LSTM	0.1171	2.48
LSTM	0.1690	5.15
BP	0.3404	7.30

**Table 2:** Electricity prediction error metrics for different models.

Electricity Consumption Prediction Model	Root Mean Squared Error	Mean Absolute Percentage Error (%)
SSA-LSTM	0.3438	3.20
LSTM	0.3555	4.12
BP	0.7691	7.40

**Table 3:** Core parameters of three carbon peaking scenarios.

	Baseline Mode	Intermediate Mode	Ideal Mode
Energy Structure Optimization	Thermal EF decreases by 1.33% annually Power EF decreases by 0.90% annually	Thermal EF decreases by 1.33% annually Power EF decreases by 0.90% annually	Thermal EF decreases by 1.33% annually Power EF decreases by 0.90% annually
Smart Heating Technology	None	Deploy IoT monitoring + dynamic regulation	Deploy IoT monitoring + dynamic regulation
Household Heat Metering	None	None	Start with 5% of users, full coverage year by year
Heat/Electricity Saving Targets	None	Initial heat/power saving rate 5%, increased by 1% year by year	Heat and power saving rates both reach 10%

## Data Availability

All data relevant to this study are available from the corresponding authors upon reasonable request.

## Abbreviations

SSA	Sparrow Search Algorithm
LSTM	Long Short-Term Memory
MAPE	Mean Absolute Percentage Error
RMSE	Root Mean Square Error
